# Longitudinal Impact of the Smoking Ban Legislation in Acute Coronary Syndrome Admissions

**DOI:** 10.1155/2017/6956941

**Published:** 2017-02-07

**Authors:** D. Abreu, P. Sousa, C. Matias-Dias, F. J. Pinto

**Affiliations:** ^1^Escola Nacional de Saúde Pública, Universidade Nova de Lisboa, Avenida Padre Cruz, 1600-560 Lisboa, Portugal; ^2^Centro de Investigação em Saúde Pública, ENSP-UNL, Avenida Padre Cruz, 1600-560 Lisboa, Portugal; ^3^Department of Epidemiology, The Instituto Nacional de Saúde Doutor Ricardo Jorge, Avenida Padre Cruz, 1649-016 Lisboa, Portugal; ^4^Serviço de Cardiologia, Hospital de Santa Maria, Centro Hospitalar Lisboa Norte, EPE, Centro Académico Medicina de Lisboa and Centro Cardiovascular da Universidade de Lisboa, Av. Prof. Egas Moniz, 1649-035 Lisboa, Portugal

## Abstract

*Background and Purpose*. The association between smoking and CV has been proved; however smoking is still the first preventable cause of death in the EU. We aim to evaluate the potential impact of the smoke ban on the number of ACS events in the Portuguese population. In addition, we evaluate the longitudinal effects of the smoking ban several years after its implementation.* Methods*. We analyzed the admission rate for ACS before and after the ban using data from hospital admission. Monthly crude rate was computed, using the Portuguese population as the denominator. Data concerning the proportion of smokers among ACS patients were obtained from the NRACS. Interrupted time series were used to assess changes over time.* Results*. A decline of −5.8% was found for ACS crude rate after the smoking ban. The decreasing trend was observed even after years since the law. The effect of the ban was higher in men and for people over 65 years. The most significant reduction of ACS rate was found in Lisbon.* Conclusions*. Our results suggest that smoking ban is related to a decline in ACS admissions, supporting the importance of smoke legislation as a public health measure, contributing to the reduction of ACS rate.

## 1. Introduction

Tobacco is now considered one of the most important public health issues and a major determinant of preventable mortality and morbidity in developed and developing countries [1, 2]. Diseases associated with tobacco consumption encompass a significant burden on individuals, societies, and healthcare systems.

Smoking affects not only active smokers but also those who are exposed to secondhand smoke in the vicinity of a smoker [3]. Passive smoking has been associated with an increase in relative risk of coronary heart disease; in some studies the exposure of nonsmokers to secondhand smoke was associated with a 25% increased risk of coronary artery disease and myocardial infarction [4]. Even short-term passive smoking appears to cause damage in the endothelial function that could immediately compromise the cardiovascular system [5].

By 2013 smoking, including secondhand smoking, was responsible for the death of approximately 12 350 people (around 11%) in Portugal [6].

The association between smoking and cardiovascular disease (CVD) has been extensively proved; however smoking is still the first preventable cause of death in the European Union [7]. Several meta-analysis showed a reduction on coronary events after the implementation of smoke-free legislation; this suggests potential to achieve vast public health benefits [8]. However fewer studies have demonstrated if the reduction effect lasted after the immediate period of the law implementation [8]. Furthermore smoking bans benefit nonsmokers and smokers. Nonsmokers benefit from the ban as they are exposed to significantly less secondhand smoke, while smokers tend to smoke less, have greater cessation success, and experience increased confidence in their ability to quit [9].

Portugal was one of the countries that signed the WHO Framework Convention on Tobacco Control [10], leading to the implementation of the most recent antismoking measure, the 37/2007 legislation implemented in January 2008 [11]. This legislation contained new framework to protect individuals from passive smoking and to encourage cutting down/stopping tobacco consumption to ensure protection against secondhand smoke [11, 12]. This law banned smoking in all enclosed public places, such as hospitals, public transportations, and workplaces. Besides this, it established further regulation for the information provided on tobacco products, packaging, and labeling, as well as further restrictions on the advertising [13].

Within the context of the growing body of consistent evidence that supports the assumption of the smoking ban being the leading cause for the decrease in the number of Acute Coronary Syndrome (ACS) events, we aim to evaluate the potential impact of the smoke legislation on the number of ACS events in the Portuguese population. In addition we aim to analyze the longitudinal effects of the smoking ban several years after its implementation and investigate trends by age, sex, and region.

## 2. Methods

### 2.1. Data

Data was obtained from the Diagnosis Related Group (DRG) National Database that collects data from all admissions into Portuguese public hospitals (mainland Portugal), holding data on primary diagnosis and some demographic variables such as sex and age, as well as the geographic region of the admission [14].

All admission cases from 2002 to 2014 were extracted and only participants with ages over 20 years old and with primary diagnosis of ACS coded in ICD 9 (international classification of disease, 9th revision) as 410.00-410.xx to identify admissions diagnosis of Acute Myocardial Infarction (AMI) and 4130 codes, to identify unstable angina, were analyzed.

Data from the National Registry of Acute Coronary Syndrome (NRACS) that gathers information on the diagnosis and treatment of ACS, being considered a surpassing source of information in Portugal [15], was used from the period of 2002 to 2010, in order to obtain the proportion of ACS patients that were current smokers. This dataset has the advantage of being integrated into the Euro Heart Survey platform and, consequently uses the Cardiology Audit and Registration Data Standards (CARDS) system. This system ensures that credible and comparable information is collected in several European countries over time as they use standardized information, in terms of both the definition and the coding of variables and in the form of measurement and collection of data. As data being validated and standardized allows for the possibility of applying and validating analysis in other larger populations, thus we are able to obtain more robust results [16].

Smoking legislation was implemented in January 2008, providing six years of data before the implementation of the legislation (January 2002 through December 2007) and seven years of data after the implementation of the legislation (January 2008 through December 2014).

The unit of analysis was monthly admission for ACS rather than an individual patient, so it was possible for a person to be counted more than once, since changes in the number of admissions were also expected to be affected by the legislation.

All data analyzed were deidentified.

### 2.2. Statistical Analysis

In order to estimate ACS crude event rates population estimates by age and sex were obtained from the National Institute of Statistics for each year and were used as denominators.

ACS crude event rates (per 100000 adults) were computed for each month, using the population of the country, restricted to adult population resident in mainland Portugal with ages over 20 years. Crude rates were calculated as the number of ACS events divided by the population.

To evaluate changes over time, we used an interrupted time series design, implementing a segmented multiple linear regression model. These models are useful when the relationship between the response and the independent variables is piecewise linear, namely, represented by two or more straight lines connected at unknown values, which are usually referred as breakpoints [17]. In this study, the segmented model was implemented in order to test whether there was a significant change in the number of ACS events after the introduction of smoke-free legislation.

The response variable was the monthly crude event rate of ACS. The impact of smoke-free legislation, defined as a change in the rate of admissions for ACS after the legislation, was assessed by including January 2008 as breakpoint in the regression. Difference between slopes, before and after the legislation, was assessed through Davies test. A month indicator variable was introduced to adjust for seasonality in ACS admissions.

All analyses were stratified by sex, age, and region. Age was grouped into two categories <65 and ≥65 years old. We further stratify data per NUTSII regions for mainland Portugal (Alentejo region, Algarve region, Midlands region, Lisbon and Tagus Valley region, and North region) in order to check for any marked reduction in regions with big cities, such as Lisbon and some cities in the North region.

Autocorrelation between month estimates was incorporated adequately into the model, with the presence of short-term autocorrelation applying a first-order autoregressive, AR (1), structure to the residuals.

Using data from the NRACS, we computed the monthly proportion of patients with ACS diagnosis that were current smokers for the period of 2002 to 2010. The proportion was obtained by dividing the number of monthly smokers by the total smokers included in the database for the study period. In order to assess if there was a significant decrease in the proportion of smokers after 2008 another segmented multiple linear regression model was implemented and Davies test was used to assess for significant changes in slopes.

Models were fitted in R version 2.3.2 software using the library segmented [18].

## 3. Results and Discussion

### 3.1. Results

A total of 190,974 cases of ACS were registered in the country (mainland Portugal), from 2002 to 2014, of which 64% were males.

**Figure 1:**
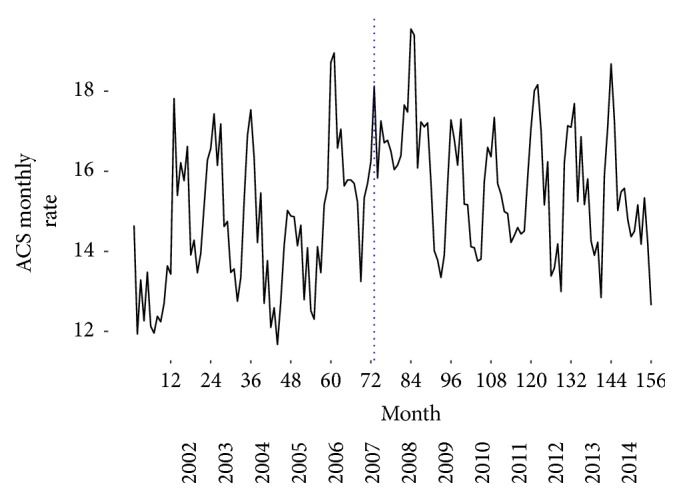
Longitudinal trends for overall monthly crude rates (per 100000 adult population) of ACS admissions from January 2002 to December 2014. Prelegislation and postlegislation periods.

We assessed crude rates (per 100000 adults) of monthly admission by ACS for the period of 2002 and 2014. Our results show a decline of ACS events by 2008, year of the law implementation, that was used as a breakpoint in the model created (Figure 1). A positive trend was observed for the years preceding the smoking ban (yearly trend of 0.004 events per 100000; 3.8%). For the period after the legislation took place, the trend observed for ACS crude rate was negative, with a decrease of 0.0018 events per 100000, that is, a decline of −1.7% by year after the legislation (Table 1). The difference between the two slopes for the trends before and after the legislation was significant (−0.006 events per 100000, *p* < 0.001) in the overall population (Table 1) with a decline of ACS events of −5.8%. We stratified the ACS events by sex, and the trends observed for the prelegislation and postlegislation periods were similar for both men and women (Table 1). Although the trends were similar, the reduction in ACS event crude rate was more marked in men (−0.0046 events per 100000; −4.8%, *p* value = 0.0002) than women (−0.0033 events per 100000;-3.2%  *p* value = 0.0002). However, the results obtained for males should be interpreted with caution as the confidence interval for the slope after the implementation of the law is wider than the one obtained for women and zero was included.

The results for age stratification show a significant reduction in ACS rate after the legislation took place for people over 65 years old (−0.014 events per 100000; −13%  *p* value < 0.001); however this finding was not verified for people under 65 years old.

Results stratified by region show that the only region with a significant decrease in ACS events after the implementation of the law was Lisbon and Tagus Valley (change in slope of −0.013 events per 100000, 12.4%, *p* value < 0.001). Although the North region also showed a trend in decrease after the implementation of the law this reduction was not significant (change in slope of −0.003 per 100000, −2.9%, *p* value = 0.168). The remaining regions did not show any significant change.

The seasonal pattern observed was consistent with that reported elsewhere [19], with higher rates of admission over the winter and lower rates during the summer.

**Figure 2:**
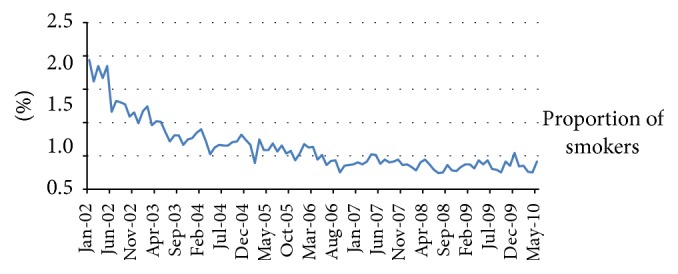
Proportion of ACS patients that are current smokers.

The analysis of the NRACS data allowed us to assess the proportion of ACS patients that were currently smokers for the period 2002 to 2010. At the time of the registry, the proportion of smokers among ACS patients has been steadily declining over the years; however the decrease observed after 2008 was not significant (*p* value = 0.997) (Figure 2).

## 4. Discussion

The mechanism by which tobacco smoke is associated with ACS has been highly studied, proving that small exposures seem to increase platelet aggregation and alter endothelial function causing other hemodynamic changes that can increase or trigger ACS events [20].

The magnitude of the effect of smoke regulations on ACS events has been found to be very broad, from studies that found no significant reduction after the smoking ban [21, 22] to studies that found very large effects from 27% to 40% reductions [23, 24]. However, these two last studies had very small sample sizes and had a very limited time after the ban (6 months).

The pooled estimate, obtained from a meta-analysis that included more than 30 studies, showed a risk reduction of 10% in ACS events after the smoking ban (95% CI 6 to 14, *p* < 0.001) [8]. Another meta-analysis study showed a pooled reduction up to 15% in ACS events after the implementation of the law [25].

Our results show that the implementation of a law to regulate smoking was associated with a decline of hospital admissions by ACS. The change in trend of ACS events from the period before the legislation to the period after the legislation was a decrease of 5.8%. Moreover the trend of ACS events for the period before the smoke legislation was increasing.

Although the effect of the reduction is not as high as some other studies, this could be explained by the fact that Portugal is among the countries with the lowest proportion of smokers in Europe (around 23%) [6, 26]. However according to the report from the Directorate-General of Health and the National Health Institute, constituting a partnership called InfoTabac, the proportion of smokers decreased by 1% in 2009, compared to 2006, but between 2009 and 2012 the smoking rate has remained stable in Portugal [10, 27], as well as for the period between 1995 and 2006 by which the prevalence of smokers was also stable [28].

On the other hand inconsistent compliance with the smoking law has been reported in few studies. The irregularities in the compliance with the law may be due to some ambiguities/gaps in the law, lack of practical definitions, and absence or delay in the effective application of penalties in case of law violations [29, 30].

A study published using Portuguese data on asthma [12] demonstrated that at least 39.6% of the sample described positive changes such as improvement of daily life activities performance, decrease in symptoms or lesser recourse to SOS medication, after the implementation of the law. From this group, 81.6% reported that since the implementation of the law, they were no longer exposed to secondhand smoke. This reinforces the hypothesis of an association between smoking ban regulation and positive health outcomes.

The seasonal pattern observed in Figure 1 was consistent with that reported in other studies [31, 32], higher rates of admission during the winter (especially around December and January) and lower rates in the summer. The higher rates of ACS during the winter could be explained by increases in blood pressure and in fibrinogen on cold days as well as the increase in infectious diseases, which are more common in industrialized countries during colder weather [33].

In January 2009, the year after the implementation of the law, a cold wave was experienced in Portugal explaining the spike in ACS rates presented in Figure 1 [34].

Bearing in mind that most of the meta-analyses studies [8, 35, 36] evidenced that longer follow-up times were needed to assess later effects of the legislation, as, in fact, the longer smoke-free policies are in place, the more pronounced are their effects on smoking behavior [37]. Thus the reduction in ACS rates observed over time in our study suggests that the effect of the legislation was sustained over time. One possible explanation for later effects is they are due to less rapidly mediated effects on atherosclerosis severity and prevalence [38].

Due to the longer duration of our study, compared to most studies, and the fact that hospital admissions were captured through a large and well-validated population database, a better delineation of trends was allowed [8, 35].

Our results show that males were associated with a greater reduction of ACS events after the legislation in declination of women. Several studies confirm these results showing more effects in male population than in female population [31, 39–42]. According to the Directorate-General of Health there is a higher rate of ACS events among men than among women; this could potentially increase power to detect an effect in men [38, 41, 43]. The fact that women present lower prevalence of tobacco consumption than men [44] could lead to lower the impact of the law, although this would not affect secondhand smoking.

The results stratified by age show a significant reduction of ACS events for older people, age over 65 years old; however no significant effect was found for people under 65 years old. The fact that older people are more vulnerable to exposure to secondhand smoking may trigger ACS events. Also it is known that arterial wall stiffness increases in subjects over 55 years. Studies performed in Mediterranean populations such as Spain and Italy showed similar effects in older subjects [42, 43, 45].

Although one of the main purposes of smoking bans is to decrease exposure to secondhand smoke, we assessed the proportion of ACS patients that reported to be current smokers using the NRACS data (Figure 2). Although the proportion of smokers with ACS diagnosis decreased from 2002 there is not a marked decrease after 2008. The proportion of smokers remained very stable between 2006 and 2010; this is consistent with the other studies. Barone-Adesi and colleagues calculated that the contribution of reduced active smoking to the reduction found in ACS was less than 1% [22]. In addition the study from Ferrante and colleagues reported that the implementation of the law did not impact smoking prevalence [46].

The findings for each region show a significant decrease only for Lisbon and Tagus Valley region. As Lisbon is the capital of country one could argue that people on bigger cities are more likely to frequent cafes, restaurants, and bars, benefiting most people from any reduction in secondhand smoking, even at low levels [21]. The North region of the country also includes big cities, so although it was not significant there was a decreasing trend in ACS events.

Although the Alentejo region is the region with higher rate in male regular smokers in mainland Portugal [26] the fact that no reduction in ACS rate was found after the implementation of the law could be related to the fact that changes in smoking behaviors may be more difficult in regions with more rural areas [47].

There are encouraging indications suggesting the effects of smoking law regulation in reducing the number of ACS events in the Portuguese population. However we recognize some limitations of the study. Like any ecological study, it is not possible to prove directly the association between the implementation of smoking law and the decline in the number of ACS events. On the other hand, by 2010 a law aiming to reduce salt in bread and making it mandatory to include information on salt content in packed food was implemented in Portugal. Although the effect of this law may be related to the declining in ACS events after 2010, the reduction in ACS rate was significant by the beginning of 2008, year of the implementation of law. Furthermore, more studies are needed to assess the effect of salt reduction legislation on ACS events.

Moreover, our study has some strengths as the availability of information on gender, age, and region which allowed us to assess the robustness of our findings among different subgroups. Also the time series method is preferred over the simpler pre- and postproportion comparison method that does not take the preintervention trend into account and also allows correction for autocorrelation [48]. The fact that we used two well validated and standardized databases is also a major strength as this allows for an easy comparison with other international studies mainly those developed in other European countries.

## 5. Conclusions

Our study extended the existing literature on public health interventions and suggests that smoking legislation is related to a decline in ACS admissions supporting the importance of smoke legislation for public health. Considering that coronary heart disease is one of the leading causes of death in Portugal, even a small reduction in the number of ACS events has important health and economic gains.

The increasing number of ACS events verified from 2002 to 2008, before the implementation of the law, may suggest that the decline of 5.8% in ACS events shown in our study is associated with the introduction of smoking legislation. It also suggests that the decline in the number of ACS events is not related to long term trends.

Our results provide encouragement for legislators and public health authorities to keep the efforts to ensure law compliance and to create new normative instruments and clinical and policy strategies to reduce the burden of ACS.

## Figures and Tables

**Table 1:** Results of multiple linear regression analyses to detect association between smoke-free legislation and monthly crude rates of ACS admissions per 100000.

	Prelegislation trend (change per month)	Change in trend in postlegislation period compared to prelegislation	Postlegislation trend (change per month)
Overall			
*β*^*∗*^	0.004	−0.006	−0.0018
IC for *β*	0.0029; 0.0055	*p* value < 0.001	−0.0033; −0.0004
Yearly change%	3.84	5.76	1.73
Males			
Β^*∗*^	0.0034	−0.0046	−0.0012
IC for *β*^*∗*^	0.0021; 0.0047	*p* value = 0.0002	−0.0028; 0.0004
Yearly change%	3.26	4.80	1.15
Females			
Β^*∗*^	0.0017	−0.0033	−0.0016
IC for *β*	0.0008; 0.0023	*p* value = 0.0002	−0.0027; −0.0004
Yearly change%	1.63	−3.17	−1.54
Age ≥ 65			
Β^*∗*^	0.0016	−0.01352	−0.0119
IC for *β*	−0.0018; 0.0051	*p* value < 0.001	−0.01625; −0.0075
Yearly change%	1.54	−12.98	−11.42
Age < 65			
Β^*∗*^	0.0011	−0.0052	−0.0483
IC for *β*	0.0008; 0.0014	*p* value = 0.2692	−0.1573; 0.0607
Yearly change%	1.06	−4.99	−4.64

^*∗*^All regression models were adjusted for seasonal effects.
